# A novel *COL4A1* variant associated with recurrent epistaxis and glioblastoma

**DOI:** 10.1038/s41439-021-00150-0

**Published:** 2021-05-14

**Authors:** Kohei Muto, Ryosuke Miyamoto, Yuka Terasawa, Yoshimitsu Shimatani, Keijiro Hara, Takumi Kakimoto, Tatsuya Fukumoto, Yusuke Osaki, Koji Fujita, Masafumi Harada, Hisanori Uehara, Yasushi Takagi, Yuishin Izumi

**Affiliations:** 1 Department of Neurology, Tokushima University Graduate School of Biomedical Sciences, Tokushima, Japan; 2 Department of Neurology, Brain Attack Center Ota Memorial Hospital, Hiroshima, Japan; 3 Department of Neurology, Tokushima Prefectural Central Hospital, Tokushima, Japan; 4 Department of Neurosurgery, Tokushima University Graduate School of Biomedical Sciences, Tokushima, Japan; 5 Division of Pathology, Tokushima University Hospital, Tokushima, Japan; 6 Department of Radiology, Tokushima University Graduate School of Biomedical Sciences, Tokushima, Japan

**Keywords:** Cerebrovascular disorders, Disease genetics

## Abstract

*COL4A1*-related disorders are characterized by a higher incidence of cerebral hemorrhage than other hereditary cerebral small vessel diseases. Accumulating data have shown broad phenotypic variations, and extracerebral hemorrhages have been linked to these disorders. Moreover, the coexistence of neural tumors has been described. Here, we report a Japanese family with a novel *COL4A1* variant, including a patient with recurrent epistaxis and glioblastoma.

*COL4A1* encodes the α1 chain of type IV collagen, which consists of six types of α chains and forms the basic framework of the basement membrane. Mutated *COL4A1* disrupts the basic structure of cerebral small vessels via a dominant-negative effect or haploinsufficiency, which results in autosomal-dominant cerebral small-vessel disease^1,2^. Approximately 60% of patients with *COL4A1*-related disorders present with diffuse white matter lesions, 50% with microbleeds, and 10–30% with hemorrhagic stroke^3,4^. The high incidence of intracerebral hemorrhage illustrates the clinical differences from other hereditary cerebral small-vessel diseases, such as cerebral autosomal dominant arteriopathy with subcortical infarcts and leukoencephalopathy (CADASIL), cerebral autosomal recessive arteriopathy with subcortical infarcts and leukoencephalopathy (CARASIL), and hereditary endotheliopathy, retinopathy, nephropathy, and stroke (HERNS)^3^. To date, more than 350 patients and 70 pathogenic and likely pathogenic variants have been reported^5^. The phenotypic spectrum of *COL4A1*-related disorders has been expanding, and accumulating data have shown that extracerebral hemorrhages may also occur^4^. Moreover, the coexistence of neural tumors has been observed^6^. Here, we report a Japanese family with hereditary cerebral small-vessel disease with a novel missense variant in *COL4A1*, including a patient who presented with recurrent epistaxis and glioblastoma.

The index patient was a 38-year-old Japanese man (Fig. 1a, patient II-1). His psychomotor development was uneventful. Multiple members of his family had a neuropsychiatric history: his mother was affected by epilepsy and juvenile dementia; his sister was affected by epilepsy, juvenile stroke, and depression; and his brother was affected by juvenile stroke. At age 5, he was admitted to a hospital due to recurrent vertigo, which disappeared within several weeks. He used to have a chronic headache without aura, nausea, and vomiting. The headache started in his early teens and disappeared by age 30. He also had a history of left scleritis.

**Fig. 1:**
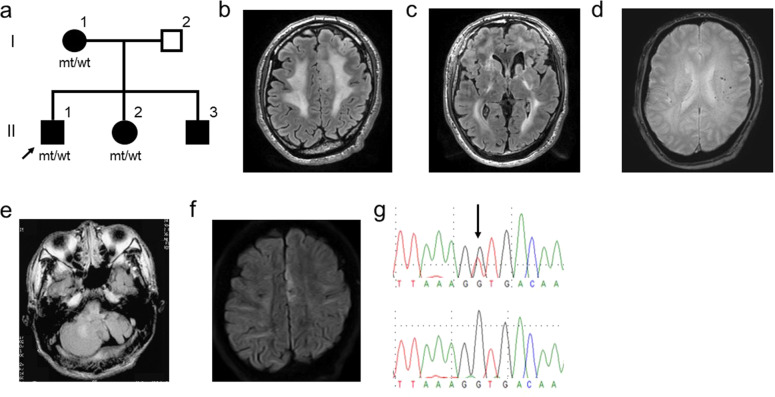
Pedigree, brain MRI of the index patient, and Sanger sequencing results. **a** Pedigree chart of the family. The arrow indicates the index patient. **b** Diffuse white matter lesions on FLAIR imaging. **c** Porencephalic enlargement of the left ventricle. **d** Scattered microbleeds on T2* imaging. **e** Left cerebellar atrophy with a cleft on FLAIR imaging. **f** Abnormal hyperintensity in the superior frontal gyrus on DWI, which corresponds to the mild hyperintensity in **b**. **g** Sanger sequencing results of the identified variant: upper panel, index patient; lower panel, control.

At age 38, he developed spasms in his right upper limb, which became generalized, and he lost consciousness. He was diagnosed with epilepsy and treated with levetiracetam and lacosamide, which markedly reduced the frequency of seizures. Brain MRI revealed bilateral white matter lesions and cerebellar abnormalities. At approximately the same time, the patient began to experience spontaneous epistaxis. Bleeding often occurred more than three times a day but could be stopped by the compression of the nasal wings; thus, he did not seek medical care for this condition. The bleeding events were apparently not associated with the seizures. He was referred to Tokushima University Hospital due to white matter lesions, epilepsy, and a complicated neurological family history that suggested hereditary small-vessel disease.

On examination, he was 189 cm tall and 87 kg in weight and had a funnel chest. He had mild hypertension. He presented with horizontal gaze nystagmus, mild spasticity in his left lower limb, and exaggerated left patellar and Achilles tendon reflexes. Otherwise, he did not have neurological abnormalities. Brain MRI demonstrated diffuse bilateral hyperintensity in the white matter and porencephalic enlargement of the left ventricle on fluid-attenuated inversion recovery (FLAIR) imaging (Fig. 1b, c) and scattered microhemorrhages on T2*-weighted imaging (Fig. 1d). At the infratentorial level, moderate hypoplasia of the left cerebellar hemisphere with a cleft reaching the fourth ventricle was observed (Fig. 1e). Hyperintensity in the left superior frontal gyrus was noted on FLAIR (Fig. 1d) and diffusion-weighted images (Fig. 1f), which was thought to be a secondary change following epilepsy. MR angiography showed no evidence of cerebral aneurysm. Blood testing showed mildly decreased hemoglobin (11.2 mg/dl) and mildly increased CK (301 U/l) levels, but the other indicators were normal. Urinalysis showed microscopic hematuria. Abdominal echocardiography revealed simple cysts in both kidneys. Ophthalmic examination showed mild cataracts, but tortuous retinal arteries and retinal hemorrhages were not observed.

The genetic study was approved by the ethics committee of Tokushima University, and the patients provided written informed consent. Genomic DNA (gDNA) was extracted from the peripheral lymphocytes. gDNA libraries were prepared using SureSelect Human All Exon V6 (Agilent Technologies, Santa Clara, CA). Sequencing was performed on 100-bp paired-end reads using a HiSeq2000 sequencer (Illumina, San Diego, CA). The reads were aligned to the human reference genome using Burrows–Wheeler Aligner software^7^. Variants were identified using GATK software^8^ and annotated using wANNOVAR^9^. All of the reported genomic locations were in GRCh37/hg19. Exome sequencing revealed a heterozygous missense variant in *COL4A1* (NM_001845.6:c.3797G>T, p.Gly1266Val), which was validated via Sanger sequencing (Fig. 1g). The variant was not registered in dbSNP Build 153, Genome Aggregation Database v2.1.1, or an in-house exome database consisting of 491 patients with various neurological diseases. The variant was predicted to be likely deleterious by PolyPhen-2 (http://genetics.bwh.harvard.edu/pph2/), pathogenic by Mutation Taster (http://www.mutationtaster.org/), and deleterious by SIFT (http://sift.jcvi.org/). The CADD Phred score (https://cadd.gs.washington.edu/) for the variant was 24.8. The variant changed conserved glycine residues within the Gly-Xaa-Yaa repeat of the collagen triple-helical domain, which is the most common substitution associated with *COL4A1*-related disorders^10^. Moreover, a different amino acid substitution at the same position, p.Gly1266Arg, was previously reported^11^. According to the 2015 guidelines of the American College of Medical Genetics and Genomics^12^, the variant was classified as being likely pathogenic. We confirmed that the patient’s mother and sister harbored the same variant via Sanger sequencing. Moreover, we obtained brain MRI scans of these two patients, which revealed diffuse white matter lesions and multiple microbleeds on FLAIR and T2*-weighted images in both (Fig. 2a–d). Taken together, we diagnosed the patient and his two affected family members with *COL4A1*-related disorders.

**Fig. 2:**
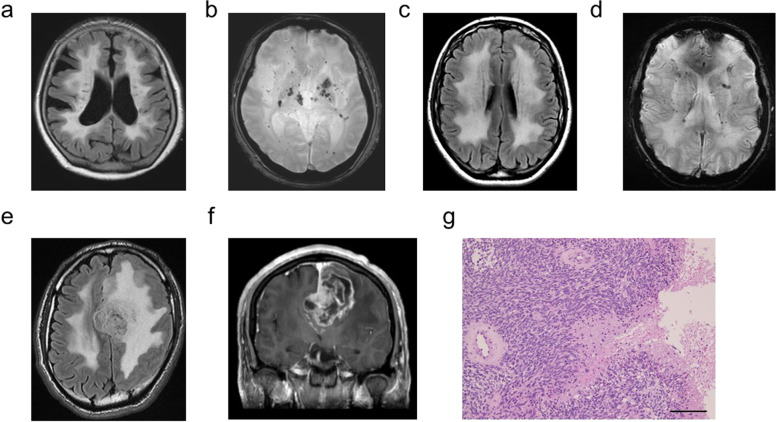
Brain MRI of the index patient’s mother and sister and the characteristics of the glioblastoma. **a**, **b** Brain MRI of the patient’s mother (**a**, FLIAR image; **b**, T2* image). Note that the patient’s mother showed moderate brain atrophy. **c**, **d** Brain MRI of the patient’s sister (**c** FLAIR image; **d** T2* image). **e**, **f** MRI characteristics of the glioblastoma in the index patient (**e** FLAIR image; **f** gadolinium-enhanced T1-weighted image). **g** The resected brain tissue stained with hematoxylin and eosin. Note the marked hypercellularity, nuclear atypia, microvascular proliferation, and necrosis. Bar = 100 µm.

The epistaxis spontaneously subsided approximately 4 months after onset. Nine months after the onset of epilepsy, he developed weakness in his right upper and lower limbs. Brain MRI revealed a mass in the left frontal lobe, which was where FLAIR and DWI hyperintensity had been observed in the previous examination. The mass expanded aggressively and showed a ring-like enhancing pattern (Fig. 2e, f). The patient subsequently underwent resection followed by radiotherapy and temozolomide. Upon pathological examination of the resected tissue, a diagnosis of glioblastoma (WHO grade IV) was made. Genetic analysis of the specimen showed a mutation in the core promoter region of *TERT* (NC_000005.9(NM_198253.2):c.-146C>T) and wild-type IDH1 R132 and IDH2 R172, which is a typical pattern observed in grade IV glioblastoma^13^.

Broad phenotypes have been linked to *COL4A1*-related disorders, ranging from catastrophic hemorrhage in the uterus to adult-onset hemorrhagic/ischemic stroke, epilepsy, and even clinically asymptomatic patients^4,5^. A wide intrafamilial variation in clinical symptoms has also been confirmed^11^. In this family with p.Gly1266Val, epilepsy, white-matter disease, microbleeds, and juvenile ischemic stroke were the core clinical features, and each phenotype occurred in multiple individuals. However, porencephaly, juvenile dementia, and depression occurred only in unique individuals. Moreover, the cardinal neurological features associated with the previously reported variant p.Gly1266Arg include infantile hemiplegia and microcephaly^11^, which were not observed in this family, supporting the previous finding of poor genotype–phenotype correlations in *COL4A1*-related disorders^5^.

Cerebral hemorrhage is an important feature in *COL4A1*-related disorders. Perinatal cerebral hemorrhage may lead to porencephaly^1,14^, which has been observed in approximately 20% of patients^4^. Of note, the index patient presented with unilateral cerebellar hypoplasia and a cleft, which were attributable to perinatal cerebellar hemorrhage^15^, in addition to a porencephalic enlargement of the left ventricle. Published data have indicated that hemorrhage may be observed in extracerebral organs. To date, six patients with retinal hemorrhages have been reported^4^. Moreover, a 30-week-old fetus was affected by hemorrhages in the thymus, liver, and adrenal glands^16^, and a 9-year-old boy developed diffuse pulmonary hemorrhage^17^. Interestingly, the index patient presented with recurrent epistaxis, which has not been reported in association with the disease. Given the expanding spectrum of the disease and the reports of extracerebral hemorrhages, we hypothesize that it might be a novel phenotype.

The development of glioblastoma in the index patient was an unexpected event. The seizure was initially considered to be a part of the symptoms of *COL4A1*-related disorders; we retrospectively found that the seizure and hyperintensity in the left superior frontal gyrus were more likely due to glioblastoma. Two cases of meningioma and one case of neuroblastoma have been reported in patients with *COL4A1*-related disorders^6^, but the authors mentioned that such common neural tumors were unlikely to be a phenotype of the disease. Consistent with this notion, glioblastoma is a common tumor in adulthood. Moreover, the presence of the *TERT* promoter mutation may support the notion that tumorigenesis in our patient was independent of the *COL4A1* variant. Nevertheless, a network analysis found that the upregulation of *COL4A1* could influence the development of glioblastoma multiforme (GBM)^18^, and the introduction of miR-29b into the GBM cell line exerted anticancer effects by inhibiting the expression of extracellular matrix genes, including *COL4A1*^19^. Considering these facts, it might be possible that the *COL4A1* variant in the index patient affected the development of glioblastoma.

In summary, we identified a two-generation family with a novel *COL4A1* variant. The index patient presented with recurrent epistaxis, which might be associated with the disease, which can present as extracerebral hemorrhages. Moreover, the development of glioblastoma in the patient might be related to the *COL4A1* variant, and further research is warranted to determine the causal relationship between *COL4A1*-related disorders and neural tumors.

## Data Availability

The relevant data from this Data Report are hosted at the Human Genome Variation Database at 10.6084/m9.figshare.hgv.3006.
